# Intestinal fungi and antifungal secretory immunoglobulin A in Crohn’s disease

**DOI:** 10.3389/fimmu.2023.1177504

**Published:** 2023-06-08

**Authors:** Meng Sun, Jingyi Ju, Hongzhen Xu, Yufang Wang

**Affiliations:** Department of Gastroenterology and Hepatology, West China Hospital, Sichuan University, Chengdu, China

**Keywords:** intestinal fungi, secretory IgA (SIgA), Crohn’s disease, antifungal immunity, mucosal immunity

## Abstract

The human gastrointestinal tract harbors trillions of commensal microorganisms. Emerging evidence points to a possible link between intestinal fungal dysbiosis and antifungal mucosal immunity in inflammatory bowel disease, especially in Crohn’s disease (CD). As a protective factor for the gut mucosa, secretory immunoglobulin A (SIgA) prevents bacteria from invading the intestinal epithelium and maintains a healthy microbiota community. In recent years, the roles of antifungal SIgA antibodies in mucosal immunity, including the regulation of intestinal immunity binding to hyphae-associated virulence factors, are becoming increasingly recognized. Here we review the current knowledge on intestinal fungal dysbiosis and antifungal mucosal immunity in healthy individuals and in patients with CD, discuss the factors governing antifungal SIgA responses in the intestinal mucosa in the latter group, and highlight potential antifungal vaccines targeting SIgA to prevent CD.

## 1 Introduction

Inflammatory bowel disease (IBD), including Crohn’s disease (CD) and ulcerative colitis, is a chronic, relapsing and incurable inflammatory disease of the intestine, with growing global prevalence in the 21^st^ century (1, 2). IBD pathogenesis involves several factors, including genetic susceptibility, environmental exposure, gut microbiota alterations, and both innate and adaptive immune responses (3, 4). Recent studies have revealed intestinal fungi as a key factor contributing to intestinal mucosal immunity and IBD development, which has raised questions regarding the specificity, functions, and mechanisms of induction of antifungal mucosal antibody responses in the gut (4–7).

In patients with IBD, structural and functional changes in the gut microbiota, including bacteria, viruses, fungi, and protozoa, disrupt the gut mucosal homeostasis, resulting in persistent and excessive immune system activation (5, 7). Many studies have focused on the role of bacteria in IBD, however, ample evidence from gut microbiota studies and studies on the immune responses to intestinal fungi suggests a potential link between fungi and IBD (7). Although fungi account for only approximately 0.1% of the total microorganisms in the gut, they have recently attracted attention for their role in IBD pathogenesis (6, 8, 9). SIgA is the most abundant antibody on the intestinal mucosal surfaces in humans and many other mammals, with approximately 3 g per day secreted into the human gut lumen (10–13). Acting as the first line of defense against pathogens, SIgA defends the intestinal epithelium by coating a substantial fraction of gut bacteria and maintaining homeostasis of the commensal microbiota (12, 14–18). Compared to healthy controls, mice and humans with reduced IgA secretion levels show an altered gut microbiota composition and increased susceptibility to IBD and other inflammatory diseases (15, 19, 20). The percentage of IgA-coated bacteria is dramatically increased in the feces of patients with IBD, particularly in patients with CD aged 17–40 years (21–23). In addition, in patients with CD, IgA-bound fecal bacterial frequencies are positively correlated with disease activity (23).

Notably, interactions between SIgA and gut bacteria may play a critical role in IBD severity (12, 23–25). However, the potential involvement of antifungal SIgA responses in CD remains largely unknown. In this review, we focus our discussion on recent advances in the understanding of intestinal fungal homeostasis and antifungal mucosal immunity in healthy and diseased (inflammatory) states. Further, we explore the roles and mechanisms of antifungal SIgA responses in the intestinal mucosa in patients with CD.

## 2 Intestinal fungal alterations in patients with CD

A study by Iliev et al. revealed that the presence of fungi, as measured by quantitative (q)PCR of fungal ribosomal DNA in the mucosa, increased from the ileum to the colon, with the highest frequencies in the distal colon (26). In the gastrointestinal tract (GIT) of humans, there are three major fungal phyla: Ascomycota, Basidiomycota, and Chytridiomycota; some research studies indicate that the colonic mucosa-associated fungal microbiota is dominated by Basidiomycota and Ascomycota (27–31), whereas other have indicated that Ascomycota are significantly more common than Basidiomycota in the human intestinal fungal microbial community (29, 30, 32–34). Using internal transcribed spacer (ITS) 2 sequencing, one study reported *Saccharomyces cerevisiae* and *Candida albicans* as the most commonly detected fungal species in stool samples of healthy subjects (35).

Numerous studies have revealed alterations in the fungal microbiota in patients with IBD. However, consensus is lacking because of the high variability of microbiomes among individuals from different areas and with different dietary preferences, and the use of different sample types and methodologies among studies (**Table 1**). Researchers have observed increased fungal diversity in colonic biopsy tissue samples or fecal samples from patients with CD in comparison to controls (36, 37). However, some recent studies of mucosa-associated microbes or stool samples reported little or no change in fungal diversity (alpha diversity) in patients with CD, but a significant increase in the global fungus load in both inflamed and non-inflamed mucosa during CD flare (29, 30). Moreover, decreased intestinal fungal diversity in stool samples from pediatric or adult patients with IBD compared to that in healthy subjects has also been reported (38, 39) (**Table 1**).

**Table 1:** Intestinal fungal alterations reported in patients with CD.

Fungi (phylum and genus levels)	Reported alterations	Microbiome samples	Methods	References
Fungal diversity	Increased in patients with CD	Colonic biopsy tissue samples, stool samples	Metagenomic 18S rDNA-based denaturing gradient gel electrophoresis, internal transcribed spacer (ITS) 1 sequencing	(36, 37)
	No significant difference (alpha diversity)	Colonic mucosa, stool samples	ITS2 sequencing and qPCR	(29, 30)
	Reduced in pediatric or adult patients with IBD	Stool samples	Deep sequencing of specific rRNA gene segments, ITS2 sequencing	(38, 39)
*Basidiomycota/Ascomycota*	Increased proportion in patients with IBD	Stool samples, mucosal lavage samples	ITS2 and ITS1 Sequencing	(32, 38)
	Decreased proportion in CD patients	Colonic mucosa, stool samples	ITS1 and ITS2 sequencing	(29, 30, 37, 40)
*Saccharomyces*	Increased proportion in CD patients	Colonic mucosa	ITS2 sequencing	(29)
	Decreased proportion in CD patients	Stool samples	ITS sequencing	(30)
*Saccharomyces cerevisiae*	Decreased proportion in active IBD patients	Stool samples	ITS2 sequencing	(38)
*Candida*	Increased proportion in CD patients	Stool samples	ITS1 and ITS2 sequencing	(30, 37)
*Candida albicans*	Increased proportion in IBD patients	Stool samples	ITS2 sequencing	(38)
*Candida glabrata*	Increased proportion in CD patients	Colonic mucosa	ITS2 sequencing	(29)
*Candida tropicalis*	Increased proportion in CD patients	Stool samples	ITS1 sequencing	(33)
*Malassezia*	Increased proportion in CD patients	Mucosal lavage samples	High-throughput ITS1 sequencing	(32)
*Malassezia globosa*	Increased proportion in CD patients	Colonic mucosa	ITS2 sequencing	(29)
*Debaryomyces*	Increased proportion in inflamed intestinal tissue of patients with CD	Ileal biopsy tissue	ITS sequencing	(41)

Some studies have revealed an increased abundance of Basidiomycota and a decreased abundance of Ascomycota, resulting in a higher Basidiomycota-to-Ascomycota ratio, in patients with CD compared to healthy controls (32, 38, 42, 43). However, other studies have indicated that the *Basidiomycota*-to-*Ascomycota* ratio is decreased in patients with CD (29, 30, 37, 40). Moreover, patients with CD have a decreased proportion of *S. cerevisiae* and an increased proportion of *C. albicans* in their gut compared to healthy subjects (30, 38). *C. albicans* can increase the production of pro-inflammatory cytokines, such as interferon (IFN)-γ, interleukin (IL)-17, and tumor necrosis factor (TNF)-α, and disrupt the gut microbiota composition to exacerbate gut inflammation (44–46). Besides *C. albicans*, *Candida tropicalis* and *Candida glabrata* are also significantly more abundant in patients with CD (29, 33). Recent studies have uncovered evidence for a strong association of *Malassezia restricta* and *Malassezia globosa* with CD, and *M. restricta* exacerbated colitis in mice in a CARD9-dependent manner (29, 32). *Debaryomyces*, particularly *Debaryomyces hansenii*, has been found to be enriched in inflamed intestinal tissues of patients with CD and to prevent colonic healing in the absence of altered Schaedler flora bacteria via the myeloid cell–IFN-γ–CCL5 axis (41) (**Table 1**).

With the rise of high-throughput sequencing, recent years have seen a steady increase in studies on the human intestinal fungal microbiota. Nevertheless, it is difficult to pinpoint the intestinal fungal alterations in patients with CD versus healthy people without differentiating the living areas and dietary preferences of study participants, the specific tissues sampled and the sampling methods used, and the methodology used to analyze the gut mycobiome. Clearly, there remain many challenges to characterizing the mycobiome. However, study findings have clearly revealed a strong association between the gut fungal microbiota and mucosal inflammation or disease activity of CD, which has drawn attention to the gut fungal microbiota composition and its possible roles in CD.

## 3 Genetic and serologic evidence for intestinal antifungal immunity in CD

Several genome-wide association studies have identified >200 loci that contribute to the risk of IBD development (47–49). These studies revealed a common polymorphism in the gene encoding caspase recruitment domain-containing protein 9 (CARD9), as one of the strongest genetic risk factors linked to CD (4, 50, 51). The *CARD9* gene is located on chromosome 9q34.3 and encodes a protein with 536 amino acids, containing an N-terminal CARD domain and a C-terminal coiled-coil domain (52–54). Expressed by myeloid cells, CARD9 is a signaling adapter protein that plays a key role in antifungal innate immunity in mice and humans, suggesting the potential links between IBD and host antifungal immunity.

As many researches reported, some CARD9 variants are closely associated with increased risk for IBD, while others are shown to be protective for IBD (55–57). The single-nucleotide polymorphism (SNP) rs4077515 in CARD9, substituting asparagine for serine at position 12 (S12N) in the protein CARD9 (CARD9^S12N^), is associated with increased expression of CARD9 mRNA and the development of CD (58, 59). A recent study reported that *M. restricta*, one of the intestinal mucosa-associated fungi significantly more abundant in patients with CD than in healthy controls, was particularly present in patients with CD carrying the *CARD9* ^S12N^ risk allele, and exacerbated colitis via CARD9 in a dextran sulfate sodium (DSS)-induced colitis mouse model (32). Conversely, CARD9 S12N Δ11 (with deletion of exon 11) fails to recruit TRIM62 to mediate CARD9 ubiquitination due to the lack of a functional C-terminal domain (57, 60). As a protective variant, it leads to negative activities of CARD9-dependent cytokine signaling including decreased TNF-α and IL-6 production (60).

**Figure 1:**
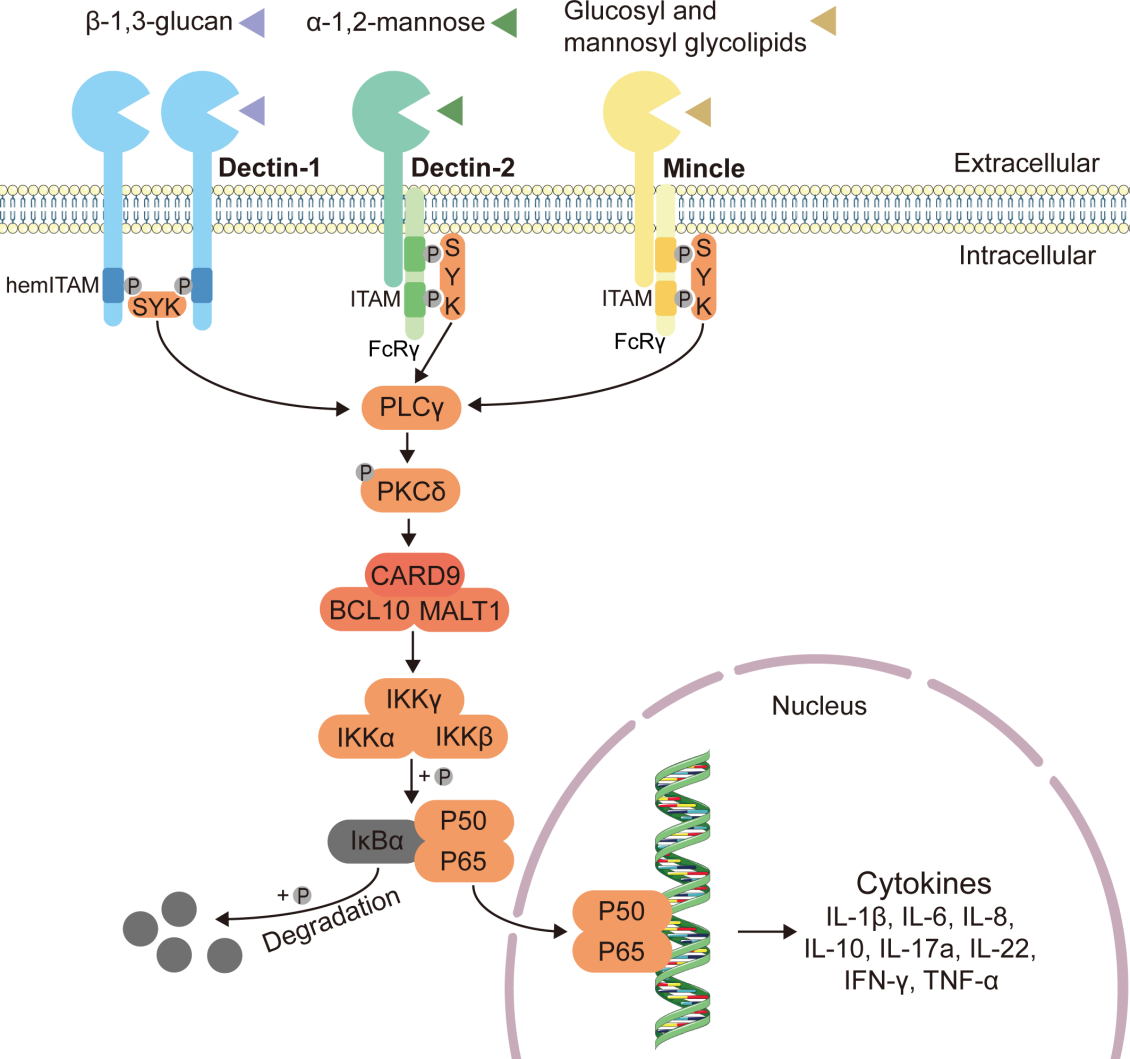
Classical innate antifungal signaling pathway through CLRs-Syk-CARD9. Expressed on myeloid cells, C-type lectin receptors (CLRs) including Dectin-1, Dectin-2 and Mincle can respectively recognize the fungal cell wall components such as β-1,3-glucan, α-1,2-mannose, glucosyl and mannosyl glycolipids, and induce intracellular antifungal signaling (26, 50, 62–64). The immunoreceptor tyrosine-based activating motif (ITAM)-coupled and the hemITAM-bearing CLRs, often considered as “activating” CLRs. Dectin-1, one of the hemITAM-bearing CLRs, can recruit spleen tyrosine kinase (Syk) directly, while the examples of ITAM-coupled CLRs, Dectin-2 and Mincle, indirectly activate Syk via the fragment crystallizable (Fc) receptor γ (FcRγ) adaptor chain (69). Syk activates phospholipase C (PLCγ), and then enhances the function of protein-kinase C (PKCδ), inducing the assembly of a CARD9–BCL10–MALT1 (caspase recruitment domain-containing protein 9–B cell leukemia-lymphoma 10–mucosa-associated lymphoid tissue lymphoma translocation protein 1) complex (65–68). The CARD9–BCL10–MALT1 complex serving as scaffolds mediates the activation of the canonical nuclear factor-κB (NF-κB) pathway (70). This mechanism activates the inhibitor of kappa B (IκB) kinase (IKK) complex, which phosphorylates NF-κB inhibitor-α (IκBα), leading to its degradation and the nuclear translocation of p65-p50 complexes (71, 72). Subsequently, the release of NF-κB dimers to the nucleus can activate gene transcription, and finally stimulate the production of related inflammatory cytokines.

As one of the major classes of the pattern-recognition receptors (PRRs), C-type lectin receptors (CLRs) can recognize the fungal pathogens and activate the subsequent antifungal immune responses in the gut (61). CLRs including Dectin-1, Dectin-2, Dectin-3 and Mincle can respectively recognize the fungal cell wall components such as β-glucan, α-mannose, glucosyl and mannosyl glycolipids (26, 50, 62–64). Upon recognizing the fungi, C-type lectin receptors recruit spleen tyrosine kinase (Syk), inducing CARD9 to couple to B cell leukemia-lymphoma 10 (BCL10) and mucosa-associated lymphoid tissue lymphoma translocation protein 1 (MALT1), ultimately leading to the activation of downstream nuclear factor kappa B (NF-κB) and production of cytokines through the CARD9-BCL10-MALT1 complex (65–68) (**Figure 1**).

Furthermore, Doron et al. presented evidence that *C. albicans* can induce the growth of germinal center (GC)-dependent IgG^+^ B cells by activating the Syk-CARD9 pathway in fractalkine/CX3C motif chemokine receptor 1 (CX3CR1)^+^ mononuclear phagocytes (MNPs), thus inducing the development of IgG antibodies to prevent systemic fungal infection (73). Moreover, intestinal fungi such as *Candida* may be recognized and phagocytosed by gut-resident CX3CR1^+^ MNPs *in vivo*. In patients with CD, there is a high correlation between a loss-of-function mutation (T280M) in the *CX3CR1* gene and a reduction in antifungal IgG responses and anti-*S. cerevisiae* antibodies (ASCA), demonstrating a connection between CX3CR1^+^ MNPs and antifungal antibodies (74, 75). Several studies have found that high serum titers of ASCA IgG and IgA, which are directed against yeast cell wall-associated mannan, are a clinical biomarker for CD (76–79). With mannoproteins expressed by cell wall, *C. albicans* was shown to express ASCA epitopes and the abundance of *C. tropicalis* was also positively correlated with the level of ASCA (4, 33, 80). Since the genus *Candida* have been described as immunogens for ASCA as the CD biomarkers, it may be responsible for an abnormal immune response in CD.

In summary, ample genetic and serologic evidence suggests a possible association between intestinal fungi, antifungal antibodies, and CD.

## 4 Structure and generation of SIgA in the intestines

### 4.1 Structure of SIgA

Different heavy and light chains assemble to create different immunoglobulins, which include five isotypes: IgG, IgA, IgM, IgE, and IgD. IgA is the most abundant immunoglobulin in humans, with a production rate of 66 mg/kg/day, and the most common antibody isotype at mucosal surfaces (81, 82). According to the amino acid composition in the hinge region and the number and locations of disulfide bonds in the heavy chain, IgA in humans is classified into two subclasses: IgA1 and IgA2. Serum IgA is comprised of 90% IgA1 and 10% IgA2, whereas IgA2 predominates in the colon (17, 83–87). The content ratio of IgA1 to IgA2 varies in different parts of the intestines, from 3:1 in the proximal small intestine to 1:3 in the colon (83, 85–88). With a shorter hinge region than IgA1, IgA2 seems more advantageous in the microbe-rich environment of the colon owing to its higher stability (86, 87, 89, 90). A recent study showed that the IgA1^+^ microbiota fraction in patients with CD was notably enriched in beneficial commensals and that local IgA2 selection of the microbiota correlated with disease activity in CD, suggesting that IgA1 has a pathogenic role in the lumen in CD, whereas IgA2 has a pathogenic role in tissues (91).

Further, IgA is expressed in different forms in different parts of the human body. Serum IgA is mostly present in the monomeric form and produced by plasma cells in the bone marrow, spleen, and lymph nodes (16, 17). In contrast, in mucosal secretions, SIgA mostly exists in a dimeric form, in which two SIgA copies are linked by a joining chain (83–85, 92). Besides stabilizing the dimeric IgA (dIgA) tail-to-tail, the joining chain also serves as a ligand for the polymeric immunoglobulin receptor (pIgR), which is an IgA and IgM transporter expressed on the basolateral surface of intestinal epithelial cells (85, 93). The resultant pIgR–dIgA complex is endocytosed and transferred across the intestinal epithelial cells, through a series of vesicles, to the gut lumen, where the extracellular portion of the pIgR is cleaved to yield a fragment termed secretory component, which then forms a complex with dIgA otherwise known as SIgA, which is released into the gut lumen (85, 94, 95).

### 4.2 Generation of SIgA through T cell-dependent and T cell-independent pathways

SIgA, locally synthesized at the mucosal surfaces, is secreted mainly by plasma cells (PCs). The SIgA-producing PCs are primarily located within the gut‐associated lymphoid tissue (GALT) including Peyer’s patches (PPs), isolated lymphoid follicles (ILFs), and mesenteric lymph nodes (MLNs) (83, 84, 96–98). SIgA-producing plasma cell can be generated via both T cell-dependent (TD) and T cell-independent (TI) pathways (13, 15, 88, 99).

TD SIgA responses typically target protein antigens and involve iterative rounds of somatic hypermutation (SHM) and high affinity selection in PPs and MLNs, where GCs are constitutively active (15, 16). Furthermore, contact between CD40 on the surface of B cells and its ligand CD40L on T cells is necessary for TD responses (88, 100). Additionally, CD4^+^ T follicular helper cells, Foxp3^+^CD4^+^ T regulatory cells, and Th17 cells can release various cytokines, such as IL-4, IL-5, IL-6, IL-10, IL-13, IL-17, and IL-21, to promote IgA responses (84, 86, 101, 102).

T cell–deficient (TCRβδ^−/−^) mice possess a microbiota-reactive IgA repertoire coating non-overlapping commensal bacterial taxa when compared with that in T cell-sufficient mice, indicating that SIgA can be produced without T cells help (15, 100, 103). TI SIgA seems to occur mainly within the gut lamina propria (LP) and ILFs (17, 88, 92). The TI SIgA response to polyvalent antigens is primarily “natural” polyreactive and shows low-affinity for commensal bacteria with little SHM (16, 103). During the TI pathway, two tumor necrosis factor family members, B-cell activating factor of the TNF family (BAFF) and a proliferation‐inducing ligand (APRIL), are responsible for stimulating class switch recombination (CSR) to IgA (86, 100). However, the contribution and function of high-affinity TD SIgA and low-affinity TI SIgA in microbiota homeostasis and infection control remain controversial (92, 94).

### 4.3 Inductive and effector immune sites in the gut

The mucosal immune system can be principally divided into inductive and effector sites (104). GALT is the site of induction of intestinal immune responses, where antigens sampled from mucosal surfaces activate naive and memory T or B lymphocytes (105). By capturing particular antigens from microfold cells or dendritic cells, B cells can generate IgA via IgM-to-IgA CSR. To synthesize IgA, the Cµ exon in the immunoglobulin heavy-chain C-region (*C_H_*) gene must be exchanged with the downstream Cα exon by activation-induced cytidine deaminase (AID) and other factors in the intestinal microenvironment (84, 88, 98, 106–108).

IgA^+^ B cells differentiate during their migration to the effector sites in the lamina propria and act as effector cells after extravasation, retention, and differentiation (84, 104, 109, 110). After the terminal differentiation of B cells to PCs, at least 80% of all PCs are located in the small intestinal LP, mainly generating dIgA which binds to pIgR, and ultimately, the formed complex is secreted into the intestinal lumen as SIgA (111).

## 5 Antifungal SIgA in patients with CD

SIgA is involved in gut microbiota maintenance. It plays multiple protective roles by preventing the adhesion of commensal bacteria to epithelial cells, neutralizing toxins and pathogens, and suppressing (not killing) fast-growing bacterial species (112–114). IgA-bound pro-inflammatory bacteria have been found to be more prevalent in patients with IBD than in healthy subjects, whereas IgA-bound commensal bacteria and probiotics levels were decreased; notably, the percentage of IgA-coated bacteria was associated with disease severity (21, 24, 115). Importantly, in addition to bacteria, intestinal fungi can also be targeted by SIgA.

By establishing a multistep flow cytometry-based technique for multi-kingdom antibody profiling, researchers recently showed that a majority of intestinal fungi were bound to luminal SIgA rather than IgG or IgM in mouse feces, independent of serum supplementation, and systemic IgA made a small contribution to the antifungal IgA repertoire (73). These findings suggest that, unlike in the blood, antifungal IgA in the intestinal tract mainly exists in the form of SIgA. Recent studies have demonstrated that, under homeostatic conditions, intestinal colonization of *C. albicans*, a potentially pathogenic fungus, induces robust SIgA responses in the human gut and oral mucosa, which is associated with elevated IgA^+^ B cell frequencies in the PPs and in the LP (73, 116–118).

As the most prevalent dimorphic fungus in the human GIT, *C. albicans* colonizes the gut as a mixture of yeast and hypha cells with varying morphologies, modes of division, occurrences, and virulence (119, 120). In general, *C. albicans* yeast is more likely to colonize the human GIT, whereas the hyphal form tends to be more virulent and invasive; therefore, the transition from yeast to invasive hyphae generally plays a central role in *C. albicans* pathogenesis (120–122). The hyphae express diverse cell type-specific virulence factors, particularly, cell surface adhesins, such as agglutinin-like protein 3 precursor (Als3), secreted aspartyl proteinases (Sap), and the Ece1-derived cytolytic toxin candidalysin (Ece1-III), which contribute to fungal pathogenesis including tissue adhesion, invasion, and damage (119, 120, 123–125).

**Figure 2:**
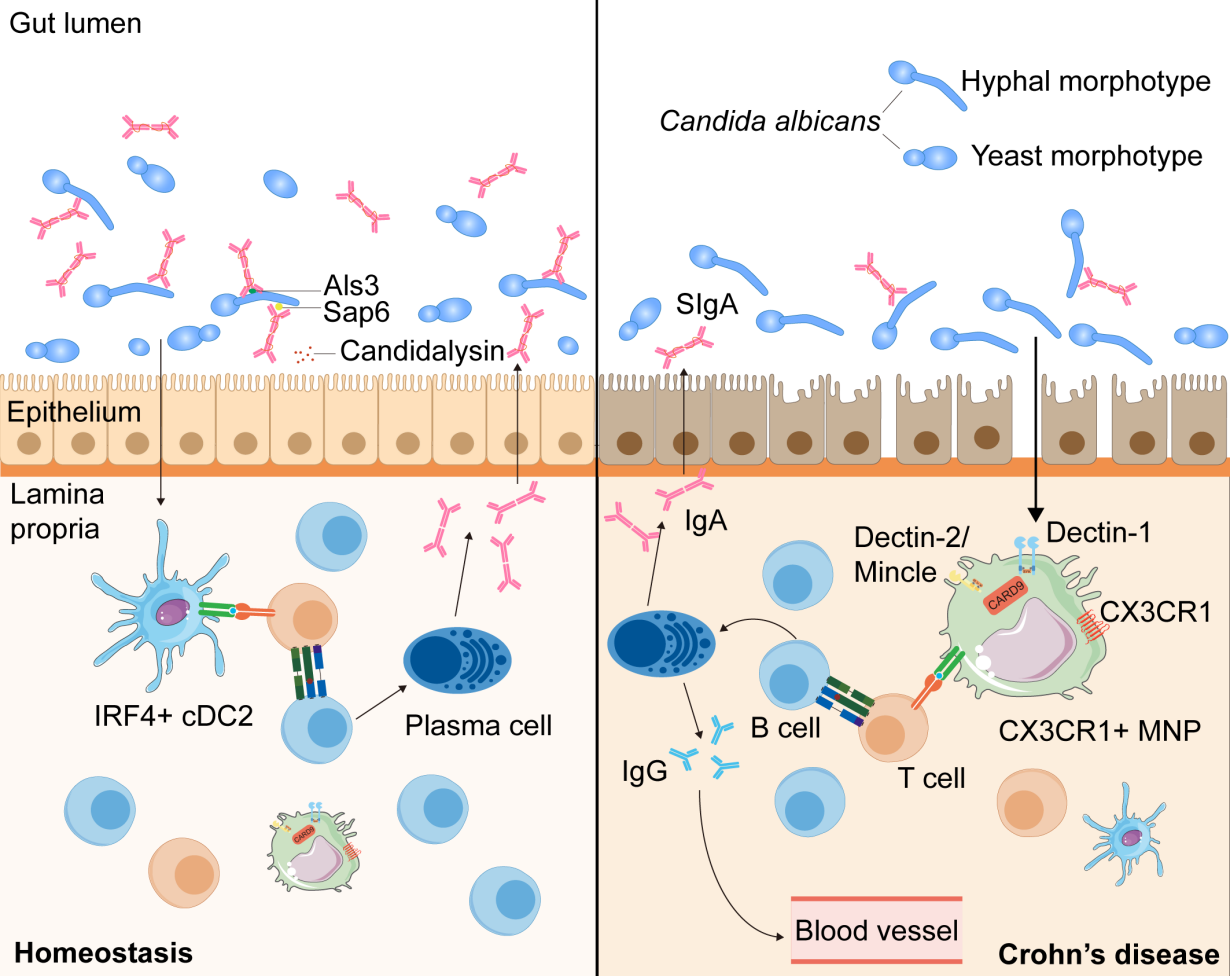
The intestinal SIgA responses targeting *Candida albicans* in the health and the patients with CD. During homeostasis, *C. albicans*, one of the commensal intestinal fungi, interacts to intestinal epithelial cells and can be recognized by IRF4^+^ cDC2s and CX3CR1^+^ MNPs, leading to the antibody CSR in mature B cells with the help of T cells (73, 116, 117). *C. albicans* can induce the growth of germinal center -dependent IgG+ B cells depending on the innate immunity regulator CARD9 and CARD9^+^CX3CR1^+^ MNPs, thus inducing the development of antifungal IgG antibodies to prevent systemic fungal infection (73). Moreover, through interaction with CX3CR1^+^ MNPs or cDC2s, the generation of SIgA targeting hyphae-associated fungal virulence factors (Als3, Sap6 and candidalysin) promotes a mutualistic relationship between the host and commensal fungi by suppressing the hyphal form of fungi (116, 117). In patients with CD, anti-*C. albicans* SIgA responses are decreased and hyphal morphotypes of *C. albicans* in the mucosa increased, which may alter the balance between the host and commensal fungi (118).

Recent work has shown that SIgA antibodies preferentially bind *C. albicans* hyphal morphotypes, targeting several hyphae-associated virulence factors (Als3, Sap6, and Ece1-III) (117, 118, 126). Consistent herewith, intervention with SIgA resulted in a reduction of *C. albicans* hyphae and promoted the growth of the yeast form (118). Additionally, studies have revealed a decrease in anti-*C. albicans* SIgA responses against hyphae-associated virulence factors and an increase in granular hyphal morphologies in the gut mucosa of patients with CD (117, 118). Together, these data suggest that antifungal SIgA, which prevents epithelial adhesion and invasion of *C. albicans* to reduce intestinal inflammation, has an important role in maintaining fungal commensalism in the gut and is dysregulated in patients with CD (**Figure 2**).

Unlike the production of systemic antifungal IgG, which depends solely on CX3CR1^+^ MNPs, anti-*C. albicans* SIgA responses are mediated by CX3CR1^+^ MNPs or CD11c^+^CD11b^+^CD103^+^ conventional dendritic cells (cDC2) via different pathways: one affecting IgA^+^ plasmablasts in the LP and the other affecting IgA^+^ B cells in the PPs (73, 74, 118, 127). Thus, intestinal fungi taken up by cDC2 and CX3CR1^+^ MNPs can induce antibody CSR in mature B cells towards the mycobiome (118). Through qPCR and flow-cytometric analysis, researchers have found that both cDC2 and CX3CR1^+^ MNPs express genes presenting antigens, but CX3CR1^+^ MNPs show higher expression of genes associated with fungal recognition, such as those encoding Dectin-1, Dectin-2, and Mincle (74). Furthermore, confocal microscopy examination has revealed that *Candida* was efficiently recognized by intestinal phagocytes *in vivo*, with >80% of all CX3CR1^+^ MNPs engulfing *Candida* (74). Moreover, a polymorphism in the coding region of the *CX3CR1* gene is strongly associated with a decrease in antifungal antibody responses in patients with CD (74) (**Figure 2**).

Overall, these results suggest that, through interaction with CX3CR1^+^ MNP or cDC2, the generation of SIgA targeting hyphae-associated fungal virulence factors promotes a mutualistic relationship between the host and commensal fungi by suppressing the hyphal form of fungi (73, 117, 118). Furthermore, in patients with CD, anti-*C. albicans* SIgA responses are decreased and hyphal morphologies of fungi in the mucosa increased, which may alter the balance between the host and commensal fungi (118). However, the detailed mechanism by which SIgA antibodies are induced by intestinal fungi and bind to the fungi in order to limit intestinal tissue damage through B or T cells remains unclear. Therefore, further studies on the mechanisms of antifungal SIgA responses, especially in patients with CD, which may provide a deeper insight into mucosal immunity and the mycobiota in the pathogenesis of CD, are urgently needed.

## 6 Potential vaccines against fungi in CD management

Currently, only a few prophylactic or therapeutic vaccines against fungal infections are in clinical trials. According to the literature, anti-*C. albicans* vaccines can be designed to target specific virulence factors in order to reduce tissue damage. The NDV-3A vaccine, which contains the N-terminal region of the Als3 protein of *C. albicans*, is the most promising and advanced vaccine tested to date (128). This vaccine has been tested for safety and efficacy against recurrent vulvovaginal candidiasis in several clinical trials; it induced robust immunologic responses with significantly elevated serum anti-Als3 IgG and IgA1 titers, and stimulation of Als3-specific production of IFN-γ and IL-17A (126, 129). The NDV-3 vaccines also induced protective responses against *Staphylococcus aureus* and *Candida auris* infections in mice (130–132). Furthermore, NDV-3 vaccination prevented intestinal damage in *C. albicans*-monocolonized mice with colitis by inducing intestinal anti-Als3 IgA responses and reducing tissue-associated *C. albicans* in the colon (117).

As discussed above, the fungal microbiota is altered in patients with CD; fungal alpha diversity, fecal fungal load, and *C. albicans* abundance are increased in these patients (43). *C. albicans* can aggravate gut inflammation by driving Th17-mediated immune responses and inducing dysbiosis of the gut microbiome (31). Collectively, these findings exemplify that SIgA antibody responses against the *C. albicans* adhesin, Als3, can alleviate *C. albicans*-associated damage during colitis, which may be useful in relieving gut inflammation in patients with CD. Consequently, novel antifungal vaccines are worth developing through further exploring the pathways via which intestinal fungi induce SIgA antibody responses in order to partially protect the intestinal mucosa from inflammation or even prevent the occurrence and development of CD by maintaining fungal commensalism in the gut.

## 7 Conclusion

In recent studies, increasing evidence indicates that intestinal fungi and SIgA responses are essential part of mucosal immune regulation in CD. The evidence discussed in this review suggests that antifungal SIgA antibodies targeting hyphae-associated virulence factors through immune interaction are a critical regulator of fungal commensalism in the gut and are essential for protecting the intestinal mucosa (73, 74, 116–118). As anti-*C. albicans* SIgA responses are dysregulated in patients with CD, an imbalance in the gut fungal community can result in overgrowth by certain fungi, leading to inflammation and pathogenic consequences (118).

Despite the increasing interest in anti-*C. albicans* SIgA responses lately, the research about other fungal species interacting with SIgA is still worth exploring. Shapiro et al. identified increased levels of IgA coating of forty-three bacterial taxa in IBD compared with controls, combining bacterial fluorescence-activated cell sorting with 16S rRNA gene sequencing (133). Through a certain degree of improvement, this promising technology, IgA-SEQ, may also have potential applications for the analysis of IgA-coated fungi in the pathogenesis of CD to identify more fungal species targeted by SIgA (133, 134). In the future, much work is required to elucidate other species that induce antifungal SIgA responses, the functions of antifungal SIgA antibodies in intestinal mucosal immunity, and the detailed mechanisms governing the induction and regulation of antifungal SIgA in the GIT in CD. These explorations will likely lead to the development of novel strategies to protect the intestinal mucosa from inflammation and maintain intestinal fungal commensalism to prevent the occurrence and development of CD, including potential vaccines.

Although the understanding of mucosal immunity and SIgA in human diseases and homoeostasis has significantly improved over the past decades, the variety and complexity of the immune response processes, functions, and mechanisms of antifungal SIgA antibodies in the intestinal mucosa of healthy subjects and patients with CD remain to be fully unraveled.

## Author contributions

MS conceived and designed the study, wrote the manuscript and finished the table and figures. MS and JJ reviewed the literature, wrote and edited the manuscript. HX analyzed articles and provided feedback on the content. YW supervised the study and reviewed and edited the manuscript. All authors contributed to the article and approved the submitted version.
